# Phytotherapy and Dietotherapy of COVID-19—An Online Survey Results from Central Part of Balkan Peninsula

**DOI:** 10.3390/healthcare10091678

**Published:** 2022-09-02

**Authors:** Nebojša Kladar, Katarina Bijelić, Biljana Gatarić, Nataša Bubić Pajić, Maja Hitl

**Affiliations:** 1 Department of Pharmacy, Faculty of Medicine, University of Novi Sad, Hajduk Veljkova 3, 21000 Novi Sad, Serbia; 2 Center for Medical and Pharmaceutical Investigation and Quality Control, Faculty of Medicine, University of Novi Sad, Hajduk Veljkova 3, 21000 Novi Sad, Serbia; 3 Faculty of Medicine, University of Banja Luka, Save Mrkalja 14, 78000 Banja Luka, Bosnia and Herzegovina

**Keywords:** phytotherapy, herbal therapy, dietotherapy, CAM, survey, coronavirus, COVID-19

## Abstract

Since the appearance of the novel coronavirus disease of 2019—COVID-19, various therapeutic approaches were attempted, with complementary and alternative medicine (CAM) taking an important place. The aim of this study was to investigate the use of CAM with the purpose of prevention or treatment of COVID-19 during the pandemics. A prospective, cross-sectional study, in the form of an on-line survey was conducted. A total of 1704 responses were collected. Among the respondents, 2.76% declared currently and 22.12% previously having COVID-19. Approximately one quarter of interviewees (23.24%) declared themselves as phytotherapy users. The most frequently used medicinal plants were garlic, ginger and chamomile. The majority of respondents stated that they expected positive effects on immune and respiratory system. Medicinal plants were frequently used, on a daily basis. The main sources of information for applied self-medication were populistic thematic literature, followed by the Internet. Approximately one-third of phytotherapy users (35.25%) consulted with a medical professional before the application of phytotherapy. Regarding dietotherapy, 41.14% of respondents reported using non-herbal dietary supplements, while only 7.16% reported specific diet. The presented results suggest that CAM is recognized and readily used as a potential alternative and complementary regimen in the fight against COVID-19.

## 1. Introduction

A pandemic of an infectious disease can sometimes be associated with high morbidity and mortality on a global scale. The first appearance of the novel coronavirus strain (named severe acute respiratory syndrome coronavirus 2—SARS-CoV-2) [1] was recorded in December of 2019 in the Chinese city of Wuhan. The respiratory disease caused by the virus was named coronavirus disease of 2019—COVID-19. A pandemic was declared on 11 March 2020 [2,3]. As of May 2022, it is estimated that more than 531 million people were infected with the causative agent, and more than 6.3 million have died [4]. In the Republic of Serbia, it is estimated that more than 2 million infections occurred, resulting in more than 16,000 death cases [5], while in the Republic of Srpska more than 112,000 infections were recorded [6].

In the short time after the declared pandemic, general preventive hygienic measures were advised as a vital mitigation strategy. Subsequently, various therapeutic regimens with conventional drugs (especially anti-infective ones) were tested [7]. Studies also pointed out the re-purposing of existing drugs, including ones previously used in the therapy of other infectious diseases, such as malaria, human immunodeficiency virus (HIV), Ebola, and influenza infections, etc., [1,8]. Currently, several types of vaccines are produced, while numerous others are still being investigated in clinical trials. Their use is now considered as the main armamentarium in the fight against the coronavirus and the corresponding disease [9].

Aside from the conventionally used drugs, patients often turn towards complementary and alternative medicine (CAM). Being defined as a set of knowledge, skills and practices based on theories, beliefs, and experiences which can be used as an addition or an alternative to conventional, mainly allopathic medicines, CAM includes acupuncture, tai chi, moxibustion, yoga, massage, and many others practices [10]. Besides them, phytotherapy or herbal medicine represents probably the most frequently encountered and socially accepted type of therapy, at the same time being the easiest for application. This type of therapy is considered a subtle transition between pharmacotherapy (therapy with conventional drugs) and traditional herbalism, as active principles are of herbal origin. Phytotherapy is defined as application of one or more medicinal plants with the purpose of prevention, treatment, or diagnosis of the disease. However, it should be pointed out that valid and rational phytotherapy only includes the use of medicinal plants within reasonably expected therapeutic aim and without harmful effects (including side effects and interactions with other drugs used by patients), in usual manners of application [11]. 

The appearance of various infectious diseases throughout the history of mankind has sparked interest in medicinal plants. As it was the case of the previous epidemics with coronaviruses, causing diseases named severe acute respiratory syndrome—SARS and Middle East respiratory syndrome—MERS, it was found that medicinal plants are of great interest for both researchers and patients using them [12,13]. Certain benefits of the use of herbs can be pointed out. First, herbal material can be treated as a valuable source of previously known secondary biomolecules, as well as a source for discovery of new ones. Modern researchers tend to investigate their pharmacokinetics and pharmacodynamics from the aspect of infectious diseases, COVID-19 included [14,15]. More frequently, medicinal plants are used as indirect and symptomatic therapy. Often added to food, or themselves being one [16], medicinal plants can also be used for strengthening the immune system. It is shown that various herbal species can act as immunomodulators through positive effects on natural killer cells, macrophages, lymphocytes, and cytokines [17]. With COVID-19 being classified as a respiratory disease, herbal preparations used in relieving the symptoms related to this type of disease are of great importance. Some of the traditionally used, and world-wide known medicinal plants possess both a high level of effectiveness and safety of use [18]. All of these, combined with the perception of phytotherapy being easily available, culturally accepted, and often cheaper than other therapeutic options, are the primary reasons for turning to herbal medicines in the midst of the COVID-19 pandemic. 

Bearing all the previously said in mind, the aim of this study was to investigate the use of phytotherapy in the prevention and treatment of COVID-19. The patterns of medicinal plants use were investigated, with a brief overview on dietary supplements and diets also applied in the prevention and treatment of COVID-19.

## 2. Materials and Methods

The research was conducted as a prospective, cross-sectional study. The survey used for questioning interviewees was specifically created for the purposes of this research. The survey was created in the Google Forms platform and placed on the web page of the Center for Medical and Pharmaceutical Investigation and Control Quality (CeMPhIC) of Faculty of Medicine Novi Sad in a separate subsection that led to this online survey. The survey was advertised through social media groups of people interested in phytotherapy, as well as in non-specific groups. Additionally, the link for the survey was distributed through official e-mails of the Faculty of Medicine, University of Novi Sad and the Faculty of Medicine, University of Banja Luka. Research was conducted during the declared COVID-19 pandemic, and surveys were obtained from December 2020 to May 2021.

The survey was written in Serbian language, in Latin script; an English translation is provided as Supplement S1. On the first page of the survey, the purpose of this research was briefly explained, and contact information were given for any further questions. The survey was organized into several sections: Sociodemographic characteristics, COVID-19, Therapy with conventional drugs, Phytotherapy, Dietary supplements (excluding ones of herbal origin), and special types of diet. Bearing in mind that this research mainly focused on medicinal plants/herbs, sub-section Phytotherapy was the most detailed, and it included list of plants previously reported to be commonly used in the investigated region (unpublished data). In the case of a negative answer in some sections (e.g., interviewee not using conventional drugs), the survey was redirected to the next section. The questions in the survey were open, semi-open or closed in type, and also included Likert scales (with 5 levels of agreement). Filling in the survey required less than 10 min. In order to evaluate the validity of the questionnaire before the research, a pilot survey was distributed to 40 people who did not participate in creating or performing the survey; the feedback on the questions was evaluated and necessary changes were made. These answers were not included in the results of this study.

As this was an online survey, no ethical approval was required. However, the interviewees gave their consent for using their answers before filling in the rest of the answers in this research. Participation was voluntary, and the anonymity of answers was guaranteed. Parents were asked to supervise filling in the survey of their underage children (˂18-year-old in the Republic of Serbia and Republic of Srpska).

All the collected data were statistically analyzed and presented using Tibco Statistica (v13.5). The results of descriptive statistics were presented as percentages and numbers, with percentages rounded up to two decimal spaces. In the case of multiple choices answers, results were presented as the number of reported uses (RU). The differences between expected and observed frequencies regarding different categories were evaluated by application of Chi-square test; values *p* < 0.05 were considered significant. Furthermore, the obtained data were analyzed by multivariate statistical technique (Multiple correspondence analysis) in order to develop a better understanding of the obtained dataset patterns of variability, as well as by data mining technique (association rules).

Multiple correspondence analysis is a multivariate statistics technique suitable for datasets containing nominal categorical data. It is one of the dimension reduction techniques that enables more efficient understanding of dataset structure by presenting it in low-dimensional Euclidian space, and all in favor of certain portion of variability loss (in this case termed inertia). The coordinates for starting variables (initially present in dataset) were computed in relation to newly formed factors (dimensions), whereas the position of evaluated variables in the space defined by factors revealed associations further to be explored.

For the evaluation of patterns of often combined medicinal plants and dietary supplements, a data mining technique (association rules) was applied. Association rules technique was based on a priori algorithm, and represents a machine learning model which analyzes data for patterns. This led to the identification of frequent if-then associations which represent the association rules.

## 3. Results and Discussion

### 3.1. Socio-Demographic Data

A total of 1704 responses were collected during the investigation period. Some of the general data regarding the interviewees are given in Table 1.

Concerning the socio-demographic characteristics of respondents of this survey (Table 1), the majority of them were women (approximately three quarters), which is consistent with the previous research of phytotherapy use in COVID-19 patients from various regions [19,20,21,22,23]. The present study also mainly included respondents with college and university degrees (4.23% and 63.38%, respectively), which is similar to previously reported results [19,21,22,23,24,25]. The majority of interviewees were employed, which is consistent with previously conducted studies of phytotherapy and various CAM uses against COVID-19 [19,20,22,23,24,25]. Regarding marital status, most of the participants were married, again in accordance with the results of other studies [20,24,25].

### 3.2. COVID-19

Regarding the COVID-19, 24.47% (417) of interviewed people declared that they had been in contact with someone who had the disease COVID-19. Only 2.76% (47) of interviewees suspected they had the disease at the time of filling in the survey. However, 22.12% (377) declared that they believe they already had COVID-19. Other studies report a lower percentage of respondents infected with coronavirus—4.1% in Iran [20], 1.6% in Hong Kong [22], and 3.42% in Saudi Arabia [25]. Approximately three quarters of all interviewed people (76.12%, 1297) declared they had not been tested for coronavirus; reasons for not engaging in testing mostly included lack of symptoms or serious symptoms (in the case of COVID-19 suspicion), lack of a need for testing, and the inability to request the testing for themselves. Out of the one quarter of interviewees who were tested, 12.50% (213) were negative for coronavirus, while only 11.38% (194) were positive.

### 3.3. Therapy with Conventional Drugs

Approximately one-third of interviewees (34.80%, 593) were using conventional therapy for various conditions and/or diseases. The most frequent reasons for conventional drug use are given in Table 2.

The opinion on the efficacy and safety of the conventionally used drugs was also investigated. The results are presented in Table 3.

Most of the people responded that they consider the therapy to be effective, with the majority agreeing or completely agreeing with the statement. Concerning the safety, interviewees generally consider their therapy to be safe; however, approximately one quarter of people using conventional therapy (24.92%) responded “I both agree and disagree”.

### 3.4. Phytotherapy

Out of the given number of interviewed people, approximately one quarter, 23.24% (total of 396 persons) declared themselves as users of phytotherapy with the purpose of prevention or treatment of COVID-19. Bearing in mind relatively high percentage of phytotherapy users reported in the present study, this may suggest that medicinal plants were mainly used in prevention of COVID-19. In previously reported studies, it was found that phytotherapy was the most frequent form of therapy (beside the conventional drugs), closely followed by the use of nutritional supplements and vitamins [24,26]. Other results contrast this, reporting vitamin supplements as the most frequent type of therapy [21,22].

A statistical testing comparing differences between phytotherapy users and non-users showed differences in some of the compared variables, namely age, level and type of education, employment status, monthly income, region of residency and marital status. Additionally, it was shown that there are differences between users and non-users of phytotherapy, regarding the use of conventional drugs and non-herbal dietary supplements. The detailed results are presented in Table 4.

The list of the most frequently used medicinal plants is given in Table 5.

Since the break-out of the pandemic, numerous medicinal plants were investigated and evaluated for their potential in the prevention and treatment of COVID-19 [27]. Among the reported medicinal plants (Table 3), the most frequently reported was garlic (*Allium sativum* L., Alliaceae). The positive effects of garlic use against COVID-19 are presumed result of immune activation, mediated by the stimulation of macrophages, lymphocytes, and natural killer cells. Additionally, it was observed that garlic can modulate cytokine secretion and synthesis of immunoglobulins [28]. Garlic was also frequently reported in other studies investigating the use of phytotherapy in COVID-19 [19,25,29]. The second most frequently used plant in this research was ginger, *Zingiber officinale* Roscoe, Zingiberaceae. This plant was previously tested in COVID-19 patients, in combination with Echinacea, where it was found that the application reduces coughing, muscular pain, and shortness of breath [30]. The third most frequently used plant was chamomile, *Matricaria recutita* L., Asteraceae. Although not clinically tested in COVID-19 patients, this plant has numerous confirmed biological and pharmacological activities (with anti-inflammatory being main one), potentially useful against this disease [31]. In vitro studies investigating potential of *Mentha* species against coronavirus demonstrated that essential oil of these species can inhibit the replication of virus in the infected cells [32].

Investigating the number of used medicinal plants, it was found that the respondents were using approximately six plants on average (mean 5.95, median 5). Furthermore, the application of association rules technique on dataset describing the medicinal plants used by respondents for the treatment or prevention of COVID-19 showed that the most commonly combined were chamomile and mint, garlic and ginger, garlic and cinnamon (Supplement S2a). Bearing in mind the previously mentioned potential of each plant, it can be assumed that plant combinations can counteract more than one step of viral infection. Combination of mint and chamomile potentially inhibits the viral replication and reduces the subsequent inflammation, while combination of garlic and ginger stimulates immune system and reduces the negative effects of coronavirus in respiratory system.

Phytotherapy users listed different reasons for its application during the COVID-19 pandemic. Most of them reported a positive effect on the immune system (367 RU). The second most frequently reported reason was a positive effect on the respiratory system (135 RU), followed by a presumed effect in the prevention and treatment of coughing (94 RU). Other reasons for phytotherapy use included: a direct effect on the coronavirus (25 RU), an antipyretic effect (19 RU), etc. In similar studies investigating the reasons for phytotherapy application, some of the main reasons for phytotherapy and other CAM uses were the prevention of coronavirus infection and the reduction of anxiety [20]. Additionally, the improvement of general well-being, and nutritional supplementation due to an inadequate diet [33] were also listed as reasons of use. However, the majority of other studies found a stimulative effect on the immune system as the main reason for the use of medicinal plants [19,22,33], as it is the case in the present study as well.

The most frequent route of administration was peroral (394 RU). Inhalation was the second most frequent route, although with much less interviewees reporting it (60 RU), followed by the application on skin and mucosa (23 RU). Most frequently, medicinal plants were used in a form prepared by the users themselves (e.g., a tea beverage) (245 RU), which is consistent with the study conducted in Bangladesh [26]. Moreover, plants were used in fresh or dried form, as part of a meal or salad (236 RU), or in a conventional form bought from pharmacies (91 RU). Moreover, interviewees used medicinal plants in a form prepared for them by someone else (e.g., essential oil, drops, tinctures etc.,) (60 RU). A study in Morocco, conducted among traditional herbalists, found that fresh herbs were most frequently used for the preparation of infusions [34]. This can potentially be explained by the convenience of preparation, bearing in mind that infusion-type beverages are frequently consumed and readily accepted by users. A study conducted in Ghana, also reported the use of raw herbal material for the preparation of herbal medicines, although finished products were reported as more frequently used [21].

Medicinal plants are frequently used. More than half of the people responded using them on a daily basis (54.11%). Besides that, 38.65% of phytotherapy users responded using plants two or more times in a week. The study in Ghana found that CAM users most frequently use the medicines on a daily basis, followed by weekly application [21]. This is consistent with the results of the presented study, where the daily or weekly use of herbal drugs was the most frequently reported regimens.

The sources of phytotherapy information were various. Most frequent sources were populistic thematic literature (books and magazines) (162 RU), followed by the Internet (152 RU). Members of the family, partners, or close friends were also frequently pointed out as sources of information (151 RU). Some of the interviewees reported consulting medical professionals (doctor, pharmacist, dentist) (134 RU), while some reported consulting herb-collectors and employees of herbal material stores (29 RU). The results of other studies reporting on the main source of information are contrasting. In some studies, the Internet and social networks are often listed as the main sources of information on phytotherapy and other CAM [20,23,24,25]. Other studies report family and friends [22,25,26], while some report health practitioners as sources of information [21].

The attitudes concerning the efficacy and safety of phytotherapy against COVID-19 were also investigated. The results are presented in Table 6.

Nearly half of the interviewees using phytotherapy considered it to be effective in the prevention of the COVID-19 disease; however, approximately one-third of people using phytotherapy responded, “I am not sure”. Other studies report similar results, with more than two-thirds of CAM users considering them to be effective in COVID-19 prevention [21]. Regarding the effectiveness of treatment of an already present COVID-19 disease, phytotherapy was perceived as less effective than prevention. One previous study found that the majority (60.7%) of CAM users considered this type of therapy not effective against the disease [24].

Considering the safety of phytotherapy, it was generally perceived as safe in the prevention of COVID-19, with approximately two-thirds of medicinal plant users declaring it safe. Similar answers were given regarding the safety of phytotherapy in treatment of an existing disease COVID-19. A study in Turkey found that respondents prefer CAM therapies precisely because of fewer side effects compared to conventional medicines [24].

Approximately one-third of phytotherapy users (30.75%) stated they have informed their doctor about the use of medicinal plants. A third of users (35.25%) consulted a medical professional before the application of phytotherapy. A different study researching CAM use in the COVID-19 pandemic report a high percentage (55%) of respondents consulting physicians prior using dietary supplements; however, this percentage was significantly lower (23.2%) for the use of medicinal plants [20]. A study in Bangladesh reported that patients prefer to seek the advice on herbal medicines from physicians rather than pharmacists [26].

Some studies compared the use of dietary supplements (both herbal and non-herbal) before and during the declared pandemic of COVID-19, and it was found that the use of these supplements increased [25]. Herbal practitioners in the United Kingdom also reported an increase in the number of patients interested in herbal therapy of COVID-19 [35]. Finally, COVID-19 was not the only reason for herbal therapy—42.50% of persons using phytotherapy against COVID-19 were also using it in the prevention or treatment of other diseases and conditions. 

### 3.5. Other Dietary Supplements and Specific Diets

Out of the total investigated number of people, a significant part (41.14%, 704) reported using non-herbal dietary supplements. The most frequently used supplements are listed in Table 7.

Dietary supplements used by the respondents reported in this research (Table 7) were also frequently reported by other research [20,22,25,26,35].

Vitamin C, potentially the most frequently mentioned among vitamins, plays an important role in the human body, especially in the immune system. Interestingly, previously published studies report only its use in treatment of COVID-19, while the data on the prevention of the diseases are lacking. A universal conclusion cannot be made, although a beneficial effect is noticed in patients with various clinical presentations of the coronavirus infection [36]. Zinc, as the second most frequently reported dietary supplement in this study, is an important microelement which inhibits viral entry and replication. Additionally, it was found in clinical studies that the use of zinc can reduce the incidence and duration of viral diseases [37]. Concerning vitamin D, the popularity of this liposoluble vitamin has dramatically increased since the declared pandemic. Bearing in mind that the deficiency of vitamin D can increase the incidence or severity of viral diseases, supplementation with this vitamin could potentially reduce the risk of infection [38]. The application of association rules technique on dataset describing the non-herbal dietary supplements used by respondents for the treatment or prevention of COVID-19 shows that the most commonly combined was vitamin C with zinc and vitamin D, (Supplement S2b). Bearing in mind the previously mentioned mechanism of activity of given mineral and vitamins, it can be assumed that these two combinations can decrease the probability of coronavirus entry into the human organism, thus reducing the risk of infection.

Concerning the changes in specific nutrition regimens, only 7.16% (122) reported being on a specific diet with the purpose of prevention or treatment of COVID-19. Most frequently, people opted for a diet with increased amounts of fresh fruits and vegetables (100 RU), a diet with organic food (24 RU), followed by a vegetarian/vegan diet (21 RU) and raw food diet (11 RU). 

It was found that a relatively small section of respondents (197, 11.56%) use both herbal and non-herbal dietary supplements. Using a previously described method (association rules technique), it was shown that in this sub-group of interviewees, garlic was most often combined with additional supplementation of vitamin C (Supplement S2c). Although results were inconclusive for vitamin C, it can be suggested that both hydrosoluble vitamin and garlic display a positive effect toward cells of the immune system, thus acting against COVID-19 in an indirect mechanism.

Additionally, an even smaller portion of the respondents is using non-herbal dietary supplements in a combination with a specific diet for COVID-19 prevention/treatment—33, 1.94%.

Contrary to the results presented here, in the Iranian population it was found that people rarely use only one type of CAM, and more frequently several of them [20]. Similarly, the use of more than one natural product was reported in the population of Saudi Arabia [19] and Hong Kong [22].

### 3.6. Multiple Correspondence Analysis

**Figure 1:**
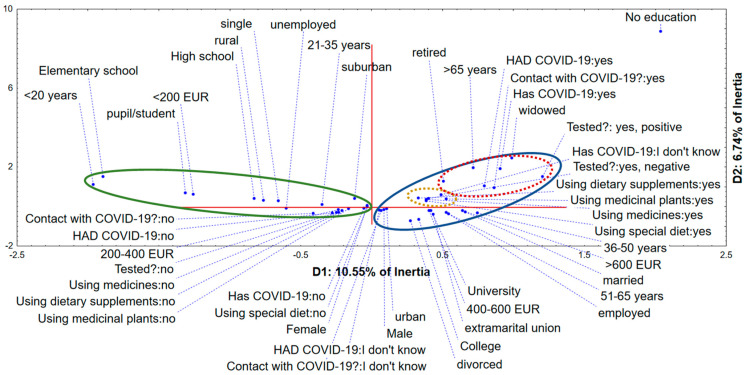
Multiple correspondence analysis—the position of recorded respondents’ answers in the space defined by the first two dimensions.

The application of a multiple correspondence analysis on variables describing the demographic characteristics of surveyed participants, as well as their habits and opinions regarding the application of medicinal plants, dietary supplements and special types of diet regarding the possible prevention/treatment of COVID-19, showed that the first two dimensions (Ds) describe around 17% of obtained dataset inertia. The computed coordinates of the recorded answers in the space defined by D1 and D2 (Figure 1) show a grouping of participants of lower monthly income and lower level of education in the negative part of D1. They represent younger categories of evaluated participants, being younger than 35 years, mostly single and living in rural or suburban areas. These participants claim that they were not in contact with COVID-19 patients, do not suspect that they have or had COVID-19, nor were tested for COVID-19. Furthermore, they are mostly not using conventional medicines for treatment of some chronic conditions, but also did not apply medicinal plants, dietary supplements, or some special types of diet for the prevention/treatment of COVID-19. On the other hand, the positive part of D1 is reserved for participants aged more than 36 years, with higher monthly income and higher level of education (graduated from colleges or universities). They are mostly married (or divorced), or living in extramarital union, inhabiting urban areas. They are not sure whether they were in contact with a COVID-19 patient or if they currently have/have previously had COVID-19. A subgroup of these participants which was tested for COVID-19 got negative results. Furthermore, this group of participants applies conventional drugs for the treatment of some chronic clinical conditions, but was also using medicinal plants, dietary supplements, and special types of diet in order to prevent/treat COVID-19. Another special subgrouping of survey participants can be observed in the positive part of D1 and positive part of D2. These participants are older than 65 years, retired, were in contact with COVID-19 patients and were tested positive for SARS-CoV-2 infection, which represents a significant concern regarding the general sensitivity of this age to COVID-19. 

These results are consistent with the results of previously published studies, where it was found that increase in age results in higher odds of using natural products or CAM [19,21]. Moreover, the use of conventional medicines increases the odds of phytotherapy use [19]. Other studies found that female participants are more likely to use various CAM [20,21,22]. A study conducted in Turkey, additionally stated that women who are married, with higher education, and higher monthly income are more likely to be users of CAM [24]. Higher education is also pointed out as a predictor of use of CAM in a study conducted in Hong Kong [22].

**Figure 2:**
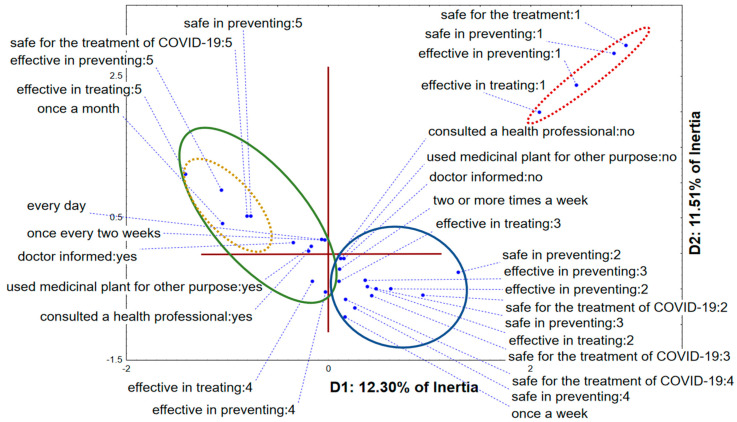
Multiple correspondence analysis—medicinal plants usage. The position of recorded respondents’ answers in the space defined by the first two dimensions.

The application of a multiple correspondence analysis on the dataset describing the opinion of the medicinal plants users regarding their efficacy and safety of application, as well as their usage habits shows that the first two dimensions describe around 24% of recorded responses variability. The position of recorded answers in the space defined by the first two dimensions (Figure 2) shows that the negative part of D1 is mostly reserved for participants recognizing medicinal plants as safe and effective in treatment and prevention of COVID-19. The level of their certainty regarding the stated subject varies from agreeing to completely agreeing, which basically separates this group in terms of D2. They are also using medicinal plants for other clinical conditions, whereas they tend to inform and consult with health professionals about the usage. On the other hand, the positive part of D1 is mostly reserved for the group of respondents being skeptical of the potential of medicinal plants application in the treatment or prevention of COVID-19, or totally denying it. They are also unsure regarding the safety of medicinal plants application, but interestingly, it seems that they apply medicinal plants more frequently (once a week; two or more times per week) than the group of respondents located in the negative part of D1. Unfortunately, this recorded higher frequency of usage is followed by total absence of reporting to healthcare professionals.

### 3.7. Strengths and Limitations of Present Study

To the best of our knowledge, this is the first study investigating the use of phytotherapy in the Republic of Serbia and the Republic of Srpska. This study had several limitations and advantages that should be listed. It was limited to literate persons, who understand the Serbian language, and additionally, bearing in mind that this survey was distributed via the Internet, and only people with web access were included. Moreover, as patients filled in the surveys by themselves, it is possible that some of them had memory bias. Although no formal validation of questionnaire was performed, a pilot survey was conducted; the feedback on the questions was evaluated, and necessary changes were made. This study was conducted before the appearance of vaccines in the Republic of Serbia and the Republic of Srpska, thus their impact on the use of phytotherapy and other CAMs was not possible to investigate. On the other hand, the study had several strengths. The survey successfully included a high number of interviewees from a relatively large geographical area of investigation. As it was presented in Table 1, diverse socio-demographic groups were included, thus it was possible to obtain more data on the use of phytotherapy and other CAMs depending on these factors. 

The data obtained in this study provide preliminary information on most commonly used herbal and non-herbal dietary supplements, their combinations, and the patterns of their use. This information is of importance in treatment of persons infected with coronavirus, as treating doctors and medical professionals should be aware of potential adverse effects and interactions of herbal and non-herbal therapy with conventional ones. For example, in this study, garlic was identified as the most frequently used medicinal herb. Bearing in mind the potential of garlic to interact with conventional drugs, doctors should be aware of the probability of patients taking this medicinal plant. Besides that, patients with milder form of COVID-19, could attribute this difference in clinical presentation precisely to use of medicinal plants and other CAMs. In the same example, further investigations are required to examine whether the intake of garlic results in shorter course of disease, less pronounced symptoms, etc., and which pattern of use brings most of benefits.

## 4. Conclusions

The results of the presented study suggest that the use of medicinal plants represents a recognized type of therapy in the fight against COVID-19. Phytotherapy is used by a significant part of interviewees, at the same time considered effective and safe for use, mainly because of the presumed positive effect on the respiratory and immune systems. Additionally, it is frequently used, in forms easily preparable and accepted by users. In order to experience the full benefit of phytotherapy, only products of the highest quality, with recognized effectiveness and safety should be used, preferably in consultation with medical professionals.

## Figures and Tables

**Table 1:** Socio-demographic data of respondents.

Question	Answer	Recorded Frequencies
gender	women	73.71% (1256)
	men	26.29% (448)
age	<20	4.58% (78)
	21–35	54.52% (929)
	36–50	27.76% (473)
	51–65	11.62% (198)
	>65	1.52% (26)
highest level of education	no formal education	0.06% (1)
	elementary school	1.17% (20)
	high school	31.16% (531)
	college	4.23% (72)
	university	63.38% (1080)
type of education	high school student in medical field	4.35% (74)
	college or university student in medical field	16.49% (281)
	medical doctor, pharmacist, dentist, or other professional in medical field	26.70% (455)
	none of the previous	52.46% (894)
employment status	pupil or student	23.88% (407)
	unemployed	7.63% (130)
	employed	65.73% (1120)
	retired	2.76% (47)
monthly income ^1^	<200 €	23.53% (401)
	200–400 €	16.32% (278)
	400–600 €	21.95% (374)
	>600 €	38.20% (651)
region of residency	Republic of Serbia	1134
	Republic of Srpska	456
	Other	114
type of settlement	urban	78.64% (1340)
	suburban	12.09% (206)
	rural	9.27% (158)
marital status	single	48.12% (820)
	married	39.85% (679)
	in extramarital union	7.28% (124)
	divorced	3.52% (60)
	widowed	1.23% (21)

^1^ Monthly income has been approximately calculated based on the currency exchange rates at the time of this research.

**Table 2:** The most frequent diseases and/or conditions which required conventional drug therapy.

Number	Reason of Use	Number of Reported Uses
1	Intermittent pain	219
2	Diseases of heart and blood vessels	138
3	Bacterial or fungal infection that required the use of antibiotics/antimycotics	136
4	Hormonal disorders (including the usage of contraceptives)	124
5	Diseases of gastrointestinal system	83
6	Diseases of respiratory system	56
7	Diseases of nervous system	26
8	Diseases of muscle, joints and/or bones	21
9	Diseases of genital and/or urinary system	19
10	Autoimmune diseases	16

**Table 3:** The opinion on the efficacy and safety of the conventional drug therapy.

Statement	Level of Agreement	Percent (and Number) of All Conventional Drug Therapy Users
I think that the therapy with the drug/drugs is effective.	I completely disagree.	0.17% (1)
	I disagree.	2.67% (16)
	I am not sure.	15.72% (94)
	I agree.	34.62% (207)
	I completely agree.	46.82% (280)
I think that the therapy with the drug/drugs is safe.	I completely disagree.	4.18% (25)
	I disagree.	5.68% (34)
	I am not sure.	24.92% (149)
	I agree.	33.61% (201)
	I completely agree.	31.61% (189)

**Table 4:** Comparison of socio-demographic characteristics of phytotherapy users and non-users.

	Variable	Phytotherapy Users	PhytotherapyNon-Users	*p*-Value ^1^
gender	women	17.61% (300)	56.10% (956)	0.500
	men	5.87% (100)	20.42% (348)	
age	<20	0.65% (11)	3.93% (67)	0.000
	21–35	9.80% (167)	44.72% (762)	
	36–50	3.70% (63)	19.19% (327)	
	51–65	8.57% (146)	7.92% (135)	
	>65	0.76% (13)	0.76% (13)	
highest level of education	no formal education	0.06% (1)	0.00% (0)	0.000
	elementary school	0.18% (3)	1.00% (17)	
	high school	5.81% (99)	25.35% (432)	
	college	1.35% (23)	2.88% (49)	
	university	16.08% (274)	47.30% (806)	
type of education	high school student in medical field	0.82% (14)	3.52% (60)	0.000
	college or university student in medical field	2.76% (47)	13.73% (234)	
	medical doctor, pharmacist, dentist, or other professional in medical field	6.16% (105)	20.54% (350)	
	none of the previous	13.73% (234)	38.73% (660)	
employment status	pupil or student	3.76% (64)	20.13% (343)	0.000
	unemployed	1.41% (24)	6.22% (106)	
	employed	17.14% (292)	48.59% (828)	
	retired	1.17% (20)	1.58% (27)	
monthly income	<200 €	4.05% (69)	19.48% (332)	0.000
	200–400 €	3.87% (66)	12.44% (112)	
	400–600 €	5.99% (102)	15.96% (272)	
	>600 €	9.57% (163)	28.64% (488)	
region of residency	Republic of Serbia	13.44% (229)	53.11% (905)	0.000
	Republic of Srpska	8.45% (144)	18.31% (312)	
	Other	1.59% (27)	5.10% (879	
type of settlement	urban	18.72% (319)	59.92% (1021)	0.480
	suburban	2.93% (50)	9.15% (156)	
	rural	1.82% (31)	7.45% (127)	
marital status	single	8.57% (146)	39.55% (674)	0.000
	married	11.74% (200)	28.11% (479)	
	in extramarital union	1.94% (33)	5.34% (91)	
	divorced	0.76% (13)	2.76% (47)	
	widowed	0.74% (8)	0.76% (13)	
use of conventional drug therapy	yes	11.03% (188)	24.06% (410)	0.000
	no	12.44% (112)	52.46% (894)	
use of non-herbal dietary supplements	yes	13.15% (224)	48.53% (827)	0.000
	no	10.33% (176)	27.99% (477)	

^1^ value *p* < 0.05 were considered significant.

**Table 5:** List of most frequently used medicinal plants.

Number	Medicinal Plant	Number of Reported Uses
1	garlic	235
2	ginger	190
3	chamomile	161
4	mint	149
5	propolis	147
6	cinnamon	120
7	turmeric	104
8	oregano	98
9	sage	92
10	green tea	89

**Table 6:** The opinion on the efficacy and safety of phytotherapy against COVID-19.

Statement	Level of Agreement	Percent (and Number) of All Conventional Drug Therapy Users
I think that the medicinal plants are effective in preventing COVID-19.	I completely disagree.	4.50% (18)
	I disagree.	8.50% (34)
	I am not sure.	30.25% (121)
	I agree.	31.75% (127)
	I completely agree.	25.00% (100)
I think that the medicinal plants are safe in preventing COVID-19.	I completely disagree.	3.75% (15)
	I disagree.	4.00% (16)
	I am not sure.	20.25% (81)
	I agree.	28.75% (115)
	I completely agree.	43.25% (173)
I think that the medicinal plants are effective in treating COVID-19.	I completely disagree.	7.25% (29)
	I disagree.	17.50% (70)
	I am not sure.	35.50% (142)
	I agree.	24.25% (97)
	I completely agree.	15.50% (62)
I think that the medicinal plants are safe in treating COVID-19.	I completely disagree.	4.25% (17)
	I disagree.	6.75% (27)
	I am not sure.	21.75% (87)
	I agree.	26.25% (105)
	I completely agree.	41.00% (164)

**Table 7:** List of most frequently used non-herbal dietary supplement.

Number	Non-Herbal Dietary Supplement	Number of Reported Uses
1	vitamin C	604
2	zinc	504
3	vitamin D	364
4	magnesium	310
5	selenium	202
6	B complex vitamins	200
7	omega-3 fatty acids	137
8	calcium	103
9	iron	90
10	vitamin E	84

## Data Availability

Not applicable.

## Supplementary Materials

The following supporting information can be downloaded at: https://www.mdpi.com/article/10.3390/healthcare10091678/s1, Supplement S1: The survey on use of medicinal plants during the COVID -19 pandemic. Supplement S2: The results of association rules technique application on dataset describing patterns of use of medicinal plants and non-herbal dietary supplements.
