# Supplementary material for: Phytotherapy and Dietotherapy of COVID-19—An Online Survey Results from Central Part of Balkan Peninsula
Source: Healthcare (Basel). 2022 Sep 2;10(9):1678. doi: 10.3390/healthcare10091678 (PMC9498599; doi:10.3390/healthcare10091678)
Supplement: Supplementary file 1 [file healthcare-10-01678-s001.zip › - Supplement S1. The survey on use of medicinal plants during the COVID -19 pandemic.pdf]

Dear Madam/Sir,

By participating in a survey called

"Use of medicinal plants during the COVID -19 pandemic"

you will be part of the research examining the use of herbal medicines and dietary supplements, as well as the details of their application.

The questions in the questionnaire refer to the **current application or application during the declared pandemic** of COVID-19, caused by the new coronavirus SARS-CoV-2.

Your information is anonymous.

Participation in the survey is voluntary. It will take you about 10 minutes to complete the questionnaire.

By filling out this questionnaire, you give your consent for us to process your data for scientific purposes.

The research is conducted by the Center for Medical-Pharmaceutical Research and Quality Control (CEMPHIC), Faculty of Medicine, University of Novi Sad.

If you have additional questions or want additional information about this research, you can contact assistant Maja Hitl (maja.bekut@mf.uns.ac.rs) or assistant professor Nebojsa Kladar (nebojsa.kladar@mf.uns.ac.rs).

Thank you for your time!

SOCIODEMOGRAPHIC CHARACTERISTICS

Gender:

1. M
2. F

Age:

1. less than 20 years
2. between 21 and 35 years
3. between 36 and 50 years
4. between 51 and 65 years of age
5. over 65 years

Highest completed level of education:

1. No education
2. Elementary school
3. High school
4. College
5. University

According to the highest completed level of education, you are:

1. high school student in medical field
2. college or university student in medical field
3. medical doctor, pharmacist, dentist, or other professional in medical field
4. none of the previous

Employment Status:

1. pupil or student
2. unemployed
3. employed
4. retired

Monthly income: *(note: income was given in local currency, and it has been approximately re-calculated to euros based on currency list at the time of this research)*

1. under 200€
2. 200-400€
3. 400-600€
4. over 600€

Region where you live:

1. Vojvodina
2. Belgrade region
3. Sumadija and Western Serbia
4. Eastern and Southern Serbia
5. Kosovo and Metohija
6. Banja Luka
7. Doboj-Bijeljina area
8. Sarajevo-Zvornik area
9. Trebinje-Foča area
10. Brcko District
11. Federation of Bosnia and Herzegovina
12. on the territory of Europe
13. on the territory of America
14. other, specify: ____________

Type of settlement in which you live:

1. urban
2. suburban
3. rural

Marital status:

1. unmarried
2. married
3. in an extramarital union
4. divorced
5. widowed

COVID-19

During the previous period, were you in contact with a person who has a confirmed COVID-19?

YES NO I DON'T KNOW

Do you suspect that you currently have COVID-19?

YES NO I DON'T KNOW

Do you suspect that you previously had COVID-19?

YES NO I DON'T KNOW

Have you been tested for the corona virus, the causative agent of COVID-19?

1. yes, the result was positive
2. yes, the result was negative
3. no

If the previous answer was NO, briefly state why you were not tested.

____

THERAPY WITH CONVENTIONAL DRUGS

Are you using / Have you used any medicine:

YES NO

Give the reason for its application: (*It is possible to circle several answers*)

1. diseases of the digestive organs (eg. high gastric acid, blood fats, liver or biliary tract diseases ...)
2. diseases of the heart and blood vessels (eg. high blood pressure, irregular heartbeat (arrhythmias), protection of blood vessels or prevention of blood clots ...)
3. diseases of the genital organs or diseases of the urinary tract (e.g. prostate diseases, inability to retain urine...)
4. hormonal disorders or use of oral contraceptives (e.g. diabetes, thyroid disease ...)
5. bacterial infection of an organ that requires / has required the use of antibiotics
6. cancer
7. autoimmune diseases
8. diseases of muscles, joints and / or bones
9. diseases of the nervous system (e.g. Alzheimer's or Parkinson's disease, depression, nervousness / tension / insomnia ...)
10. respiratory diseases (e.g. asthma or bronchitis ...)
11. intermittent pain (e.g. headache, toothache, menstrual pain ...)
12. other, specify: _____________

On a scale of 1 to 5, rate how much you agree with the following statements:

	I totally disagree	I mostly disagree	I don't know	I mostly agree	I totally agree
I think that the therapy with the drug / drugs I use is effective.	1	2	3	4	5
I think that the therapy with the drug / drugs I use is safe.	1	2	3	4	5

PHYTOTHERAPY

Have you used any medicinal plant for the prevention or treatment of COVID-19: YES NO

In the list of medicinal plants, indicate one / more that you use / have used or add a plant or herbal preparation: (*It is possible to circle several answers*)

1. aloe vera
2. chokeberry
3. artichoke
4. garlic
5. basil
6. cranberries
7. ivy
8. cinnamon
9. echinacea
10. ginkgo biloba
11. goji berry
12. common yarrow
13. Iceland moss
14. sweet flag
15. cowslip primrose
16. chamomile
17. hemp
18. nettle
19. turmeric
20. yellow gentian
21. linden
22. breckland thyme
23. lemon balm
24. cat's claw
25. mint
26. noni
27. oregano
28. coltsfoot
29. propolis
30. rtanj tea
31. marshmallow, white or black
32. wild garlic
33. common thyme
34. green tea
35. black elder
36. rosehip
37. shiitake
38. sage
39. ginseng
40. ginger
41. other, specify: ____________ ____________ _____

State the reason for its / their use in the prevention or treatment of COVID-19: (*It is possible to circle several answers*)

1. direct action on coronavirus, the causative agent of COVID-19
2. positive effect on the respiratory system
3. cough prevention or treatment
4. lowering fever
5. positive effect on immunity
6. other, specify: _________

How do you use the medicinal plant / plants for this purpose: (*It is possible to circle several answers*)

1. by drinking and / or eating
2. application to the skin and / or mucous membranes
3. by inhalation
4. application on / in the genitals and / or colon (vaginal / anal)
5. other, specify: ____

In what form do you use the medicinal plant / plants for this purpose: (*It is possible to circle several answers*)

1. as a fresh or dried plant, in powder, or in salad
2. in a form which I have independently prepared by an appropriate procedure, e.g. tea, drops
3. in a form prepared for me by someone else by an appropriate procedure, e.g. oil, tincture
4. as a medicinal form purchased at a pharmacy, e.g. tablets, capsules
5. other, specify: ____

On average, how often do you use a medicinal plant/plants or dietary supplement for this purpose:

1. every day
2. two or more times a week
3. once a week
4. once every two weeks
5. once a month

How did you find out about the use of medicinal plants for this purpose: (*It is possible to circle more answers)*

1. from a close person (family member, partner or friend)
2. from a doctor, pharmacist, dentist or healthcare associate
3. from herbalist, in herbal or health food stores
4. through books or magazines about medicinal herbs
5. via the Internet
6. through advertising or promotional material on television, in newspapers or in flyers
7. other, specify: _____________________

On a scale of 1 to 5, rate how much you agree with the following statements:

	I totally disagree	I mostly disagree	I don't know	I mostly agree	I totally agree
I think that medicinal plants are effective in preventing COVID-19.	1	2	3	4	5
I think that medicinal plants are effective in treating COVID-19.	1	2	3	4	5
I think that medicinal plants are safe in preventing of COVID-19.	1	2	3	4	5
I think that medicinal plants are safe for the treatment of COVID-19.	1	2	3	4	5

Is your doctor informed that you are using/have used the medicinal plant/plants for the prevention or treatment of COVID-19:

YES NO

Have you consulted a doctor / pharmacist / dentist before using the medicinal plant/plants that you have decided to take for the prevention or treatment of COVID-19:

YES NO

In addition to the previously mentioned plant / plants for the treatment / prevention of Covid 19, have you used any other medicinal plant for any other purpose?

YES NO

DIETARY SUPPLEMENTS AND DIET

Are you using/have you used any of the dietary supplements to prevent or treat COVID-19:

YES NO

Indicate which dietary supplements you have used: (*It is possible to circle several answers*)

1. vitamin C
2. B complex vitamins
3. vitamin A
4. vitamin D
5. vitamin E
6. vitamin K
7. iron
8. selenium
9. zinc
10. calcium
11. magnesium
12. other minerals
13. protein supplements
14. supplements of omega-3 fatty acids
15. other, specify: _______________________

Are you using/ have you used any special type of diet to prevent or treat COVID-19:

YES NO

Indicate what type of diet it is/was: (*It is possible to circle several answers*)

1. vegetarian or vegan diet
2. eating organic food
3. eating raw food
4. diet with increased amount of fruits and vegetables
5. gluten-free diet
6. diet adapted to diabetes
7. diet adapted to high blood fats
8. diet adapted to lactose intolerance (milk and dairy products)
9. other, specify: _________________________
