# Supplementary material for: Phytotherapy and Dietotherapy of COVID-19—An Online Survey Results from Central Part of Balkan Peninsula
Source: Healthcare (Basel). 2022 Sep 2;10(9):1678. doi: 10.3390/healthcare10091678 (PMC9498599; doi:10.3390/healthcare10091678)
Supplement: Supplementary file 1 [file healthcare-10-01678-s001.zip › - Supplement S2. The results of association rules technique application on dataset describing patterns of use of medicinal plants and non-herbal dietary supplements..pdf]

The results of association rules technique application on dataset describing:

a) patterns of medicinal plants application (min. support = 15.0%, min. confidence = 15.0%, min. correlation = 50.0%; max. size of body = 10, max. size of head = 10).

Body	==>	Head	Support (%)	Confidence (%)	Correlation (%)
chamomile	==>	mint	24.75	61.49	63.92
mint	==>	chamomile	24.75	66.443	63.92
garlic	==>	ginger	32.50	55.32	61.52
ginger	==>	garlic	32.50	68.42	61.52
garlic	==>	cinnamon	24.25	8	57.76
cinnamon	==>	garlic	24.25	80.83	57.76
mint	==>	garlic, chamomile	17.25	46.31	55.16
garlic, chamomile	==>	mint	17.25	65.71	55.16
chamomile	==>	garlic, mint	17.25	42.86	54.11
garlic, mint	==>	chamomile	17.25	68.32	54.11
garlic	==>	chamomile	26.25	44.69	53.98
chamomile	==>	garlic	26.25	65.22	53.98
garlic	==>	mint	25.25	42.98	53.97
mint	==>	garlic	25.25	67.78	53.97
ginger	==>	cinnamon	19.25	40.53	50.99
cinnamon	==>	ginger	19.25	64.17	50.99
garlic	==>	oregano	19.00	32.34	50.08
oregano	==>	garlic	19.00	77.55	50.08

b) patterns of non-herbal dietary supplements application (min. support = 25.0%, min. confidence = 25.0%, min. correlation = 60.0%; max. size of body = 10, max. size of head = 10)

Body	==>	Head	Support (%)	Confidence (%)	Correlation (%)
vitamin C	==>	zinc	63.91	74.17	81.19
zinc	==>	vitamin C	63.91	88.88	81.19
vitamin C	==>	vitamin D	46.93	54.47	70.16
vitamin D	==>	vitamin C	46.93	90.38	70.16
zinc	==>	vitamin D	40.94	56.94	67.00
vitamin D	==>	zinc	40.94	78.84	67.00
vitamin D	==>	vitamin C, zinc	37.94	73.07	65.87
vitamin C, zinc	==>	vitamin D	37.94	59.37	65.87
vitamin C	==>	magnesium	40.37	46.85	65.40
magnesium	==>	vitamin C	40.37	91.29	65.40
zinc	==>	vitamin C, vitamin D	37.94	52.77	65.32
vitamin C, vitamin D	==>	zinc	37.94	80.85	65.32
vitamin C	==>	zinc, vitamin D	37.94	44.03	63.88
zinc, vitamin D	==>	vitamin C	37.94	92.68	63.88
magnesium	==>	zinc	33.95	76.77	60.21
zinc	==>	magnesium	33.95	47.22	60.21

c) patterns of combined application of medicinal plants and non-herbal dietary supplements (min. support = 15.0%, min. confidence = 15.0%, min. correlation = 70.0%; max. size of body = 10, max. size of head = 10)

Body	==>	Head	Support (%)	Confidence (%)	Correlation (%)
vitamin C	==>	zinc	68.75	78.17	83.41
zinc	==>	vitamin C	68.75	89.01	83.41
vitamin C	==>	vitamin D	56.69	64.46	77.02
vitamin D	==>	vitamin C	56.69	92.02	77.02
vitamin D	==>	zinc	52.23	84.78	75.72
zinc	==>	vitamin D	52.23	67.63	75.72
vitamin D	==>	vitamin C, zinc	48.66	78.98	74.76
vitamin C, zinc	==>	vitamin D	48.66	70.77	74.76
zinc	==>	vitamin C, vitamin D	48.66	63.00	73.53
vitamin C, vitamin D	==>	zinc	48.66	85.82	73.53
vitamin C	==>	vitamin D, zinc	48.66	55.32	71.79
vitamin D, zinc	==>	vitamin C	48.66	93.16	71.79
vitamin C	==>	garlic	50.89	57.86	71.23
garlic	==>	vitamin C	50.89	87.69	71.23
chamomile	==>	mint	28.57	65.30	70.12
mint	==>	chamomile	28.57	75.29	70.12
